# Supplementary material for: Association among Vitamin D, Retinoic Acid-Related Orphan Receptors, and Vitamin D Hydroxyderivatives in Ovarian Cancer
Source: Nutrients. 2020 Nov 19;12(11):3541. doi: 10.3390/nu12113541 (PMC7699234; doi:10.3390/nu12113541)
Supplement: Supplementary file 1 [file nutrients-12-03541-s001.pdf]

Association between vitamin D, retinoic acid-related orphan receptors and vitamin D hydroxyderivatives in ovarian cancer

Anna A. Brożyna [1,*] ORCID ID 0000-0002-3195-9965, Tae-Kang Kim [2] ORCID ID 0000-0003-1394-4134, Marzena Zabłocka [3], Wojciech Jóźwicki [4] ORCID ID 0000-0001-9311-1604, Junming Yue [5], Robert C. Tuckey [6] ORCID ID 0000-0002-5022-4729, Anton Jetten [7] ORCID ID 0000-0003-0954-4445 and Andrzej T. Slominski [2,8,*] ORCID ID 0000-0002-3792-8940

Table S1. Summary of RORα expression in ovarian cancer samples.

Sample	Cytoplasmic RORα [mean A.U.]	Nuclear RORα [mean A.U.]
Normal ovary [=5]	90.0	168.0
Primary cancer [n=15]	27.7	22.4
G1	5.0	23.8
G2	25.3	20.2
G3	44.4	26.7
Metastatic cancer [n=4]	23.8	6.8

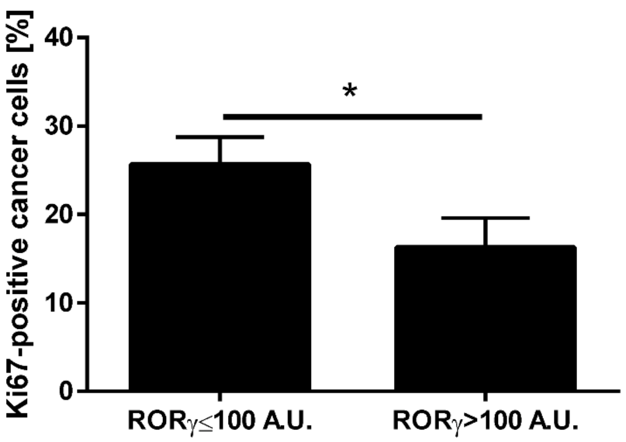

Figure S1. The relationship between nuclear RORγ and percentage of Ki-67-positive cells in central parts of the tumor, defined as the older part of the tumor, with no signs of dynamic growth [60]. Statistically significant differences are denoted with asterisks as determined by Student's t-test (*$p < 0.05$).
